# A Preliminary Investigation into Effects of Linguistic Abstraction on the Perception of Gender in Spoken Language

**DOI:** 10.1007/s12144-014-9224-7

**Published:** 2014-05-18

**Authors:** A. B. Siegling, Michelle Eskritt, Mary E. Delaney

**Affiliations:** 1 Division of Psychology and Language Sciences, University College London, WC1H 0AP London, UK; 2 Mount Saint Vincent University, Halifax, Canada

**Keywords:** Gender, Spoken language, Stereotypes, Linguistic abstraction, Linguistic category model

## Abstract

We investigated the role that linguistic abstraction may play in people’s perceptions of gender in spoken language. In the first experiment, participants told stories about their best friend and romantic partner. Variations in linguistic abstraction and gender-linked adjectives for describing their close others were examined. Participants used significantly more abstract language to describe men compared to women, possibly reflecting a gender stereotype associated with the dispositionality factor of linguistic abstraction. In a second experiment, a new group of participants judged the gender of the protagonists from the stories generated in Experiment 1, after the explicit linguistic gender cues were removed. Consistent with the dispositionality factor, linguistic abstraction moderated the effects of the gender stereotypicality of the context (masculine, feminine, or neutral) on participants’ gender judgments. Discussion focuses on the implications of the results for the communication of gender stereotypes and the effects of linguistic abstraction in more naturalistic language.

Although Western culture seeks to become gender-egalitarian and societal disapproval of gender stereotypes is becoming more widespread, progress has been slow. More subtle forms of sexist practice, in particular, still persist (Holmes 2005) and many people continue to express gender stereotypes in implicit ways, which has been referred to as *modern sexism* (Swim et al. 1995). Language, especially interpersonal discourse, is one medium through which gender stereotypes are transmitted implicitly (e.g., Harasty 1997; Lenton et al. 2009; Ochs 1992). However, investigations of how gender is represented in our language and, perhaps more importantly, how such language-mediated gender distinctions are perceived by recipients have largely concentrated on the use of explicitly gendered terms, which tend to be overtly sexist. Examples include the generic pronoun *he* to refer to any person, using job titles ending in *man*, or the asymmetry of titles as in using *Mrs.* or *Miss* to indicate women’s marital status (Speer 2002).

Consistent with the Sapir-Whorf hypothesis that language can influence thought (e.g., Whorf 1956), evidence suggests that the use of explicitly gendered terms is related to how we process and perceive information. For example, the use of the generic words *he* and *man* was found to elicit gender-biased thinking, specifically male imagery, in children and adults (Henley 1989). Similarly, American undergraduates were more likely to think of female characters when reading sentences involving *he/she* or *they* than for sentences containing the generic *he* (Khosroshani 1989). Ochs (1992) argued that in the English language there are few such direct indices of gender (beyond *he/she*, *Mr./Miss/Mrs.*, etc.) and instead gender is frequently indexed indirectly. For example, some occupations are associated with a particular gender regardless of whether or not the occupation is explicitly gender-linked by adding *man* to the end of the title (Lassonde and O’Brien 2013; Reynolds et al. 2006).

Gender enactment is multifaceted and more subtle, going beyond simple practices like the use of gender-inclusive language to include practices such as drawing attention to gender or one particular gender either directly or indirectly (Hopper and LeBaron 1998). For example, Sunderland (2002) examined a selection of parenting manuals to see how fathers were portrayed. She noted that fathers played an almost optional role in parenting with little reference to them and, when they were discussed, they were frequently portrayed as “bumbling assistants,” “line managers,” and/or “baby entertainers.” However, beyond semantic content as an indirect way of indicating gender, it is less clear whether there are specific word categories (like personal pronouns) that are not explicitly gender-linked, but nonetheless used to indicate gender implicitly. Language of this kind may also influence recipients’ perception of information in a gender-biased fashion.

## Linguistic Categories, Abstraction, and Cognitive Inferences

Interpersonal verbs and adjectives comprise the most commonly used linguistic categories in person and behavior descriptions (Semin and Fiedler 1988). One of these categories, adjectives, encompasses a few words that are explicitly gender-linked (e.g., *masculine*, *feminine*; *male*, *female*) as well as a wider range of implicit, albeit stereotypically gender-linked terms. Implicitly gender-linked adjectives reflect traditional gender-role norms and personality traits on which men and women are expected to differ (Bem 1974; Williams and Bennett 1975). For example, men are stereotypically assumed to be strong and aggressive, whereas women are thought to be more emotional and passive.

Interpersonal linguistic categories vary on a concreteness–abstractness dimension, ranging from neutral descriptions of concrete actions (e.g., *to kiss, to stare*) to psychological states (e.g., *to like, to envy*) to more abstract qualities, such as traits or dispositions (e.g., *reliable, aggressive*; see Semin and Fiedler 1988). The use of interpersonal verbs and adjectives in person descriptions has social-cognitive implications, as people make different attribute inferences based on the level of linguistic abstraction (e.g., Brown and Fish 1983; Fiedler and Semin 1988).

Linguistic abstraction can reflect communicators’ thinking, and contribute to recipients’ inferences in two different ways, indicating *dispositionality* and *event instigation* (Semin and Marsman 1994). Research regarding dispositionality indicates that individuals described in more abstract interpersonal language are seen as stable in that characteristic, regardless of context (Semin and Fiedler 1988). For example, describing someone as *kind* is typically interpreted as a trait that will persist and reveal itself in future situations. In contrast, the use of more concrete interpersonal verbs shifts the level of explanation from enduring personal or group characteristics to contextual features in accounting for particular behaviors. If someone is described as having *given money to a charity*, the likelihood of this behavior occurring again is seen as less predictable compared to *a generous supporter of charities*. Although research in this area has focused on the behavior of individuals rather than groups, it would seem logical that the same principle (i.e., of characteristic behavior being described more abstractly) applies to the behavior of groups. Thus, if people perceive one gender’s behavior as more stable or predictable than the other gender’s behavior (i.e., due to a gender stereotype), then this may be reflected in a direct association between linguistic abstraction and gender in person descriptions and perceptions.

The second type of inference, event instigation, focuses on who initiated the event being described. Specifically, the use of concrete action verbs (e.g., X *compliments* Y) also tends to be interpreted by listeners as the actor or sentence subject initiating events, whereas the use of the more abstract state verbs (e.g., X *likes* Y), which describe feeling states, tends to make the object in the sentence the causal agent (e.g. Brown and Fish 1983; Fiedler and Semin 1988). Thus, concrete action verbs assign the protagonist an active role, whereas abstract state verbs depict the protagonist as being more passive. This type of inference may also influence people’s use of linguistic abstraction in describing groups, such as men and women; a gender stereotype that one gender is more passive than the other gender could lead people to describe individuals of this gender in more abstract language. Research aiming to demonstrate a direct relationship between gender and linguistic abstraction, reflecting a gender stereotype implicit in either of the two factors of linguistic abstraction, would need to demonstrate that this link is independent of the more overtly gender-linked adjectives.

A third mechanism through which linguistic abstraction may be linked to gender is as a function of men’s and women’s actual behavior, relative to the behavior one can expect from them based on gender-role norms. Maass et al. (1995) demonstrated that both individual and group behavior that is consistent with expectations or stereotypes is described more abstractly than behavior that violates expectations. This phenomenon has been referred to as the *linguistic expectancy bias* (LEB; Wigboldus et al. 2000) and can be attributed to the greater stability and predictability of expected information. In consideration of the LEB, linguistic abstraction may also vary with gender to reflect men’s and women’s relative conformity to gender-role norms. One gender may be more likely to conform to gender-role norms than the other, leading people to describe members of this group in more stereotypical contexts and, hence, use more abstract language. This possibility implies that linguistic abstraction varies with individual differences in conformity to gender-role norms, with conforming individuals being described more abstractly than less conforming persons.

## Effects of Linguistic Abstraction on the Recipient

Building on the above findings, Wigboldus et al. (2000) asked participants to provide examples of situations where a male and a female friend acted in either a stereotypically consistent or stereotypically inconsistent manner, relative to gender-role norms. Recipients reading the gender-stereotypical descriptions were more likely to attribute the behavior to stable^1^ and enduring traits than stereotypically-inconsistent descriptions, which they were more likely to attribute to situational factors. Importantly, linguistic abstraction was found to mediate the effect of expectation consistency (i.e., relative to group-level stereotypes) on recipients’ dispositional attributions; recipients’ inferences about the person described varied as a function of the level of linguistic abstraction used by the communicator, with more abstract language reflecting greater stereotype consistency of descriptions and increasing the likelihood of dispositional attributions. However, this investigation did not fully control the effects of shared cultural stereotypes, as recipients knew the stereotypes implicit in the messages they were reading. That is, speakers were asked to describe events in which either a male or female friend acted in either a stereotypically masculine or feminine way.

In a later study, the same researchers observed that linguistic abstraction influenced inferences about *individuals* (i.e., their personalities), who were unknown to the recipients, in the same way (Wigboldus et al. 2006). Transmitters were asked to describe an event where a friend acted expectedly and a scenario where this friend acted unexpectedly, relative to the friend’s personality. Communicators described behaviors of their friends in expected events in more abstract language than behaviors in unexpected events. Again, recipients were more likely to attribute behavior to the person’s personality when the behavior was described in abstract language, whereas behavior described in more concrete interpersonal language was judged to be influenced more by the situation; linguistic abstraction mediated the effect of the level of expectancy on dispositional attributions. Together, these studies provided strong evidence that linguistic abstraction can influence people’s perception of information.

A strength of the research by Wigboldus et al. (2000, 2006) was the reliance on participants’ own written language, which lends a more naturalistic quality to the data and, thus, enhances the potential real-world relevance of the findings. Prior research on the role of linguistic abstraction in communicating about groups focused on participants’ interpretation of experimenter-produced written language, usually in the form of simple, isolated sentences (e.g., Maass et al. 1995; Semin and Fiedler 1988). While this is a valuable direction to pursue, participants were cued for role expectations in both studies, being asked explicitly to describe others in expected and unexpected ways (Wigboldus et al. 2000, 2006). This procedure, although useful in demonstrating LEB effects on recipients, does not reveal much about how linguistic abstraction is used in natural language without any directions for describing others imposed on the speaker. Further, it is unclear how linguistic abstraction as it appears in natural language might influence the recipient in making inferences about the protagonist.

In natural language, it may not be apparent relative to which general attribute (gender, ethnicity, social class, personality, etc.) linguistic abstraction is used to indicate how expected the information communicated is for the person being described. Linguistic abstraction could be concrete because the protagonist did not conform to gender-role norms, act in line with his or her personality, or behave according to cultural customs in the event described. For various reasons (e.g., instructions, biases, and the context of information presented), the recipient may then erroneously use linguistic abstraction to make inferences about some other general attribute of the person—one that is unrelated to linguistic abstraction, because the speaker used a different attribute as the reference point. When the recipient assumes an inappropriate reference point for linguistic abstraction, as is possible in natural language, LEB effects may not be “picked up” by the recipient in a clear-cut way. To our knowledge, no study has examined the effects of linguistic abstraction in more natural language without requesting, and, thus, cuing for particular norm-referenced descriptions, such as gender stereotypes. Further, spoken language is more spontaneous, less carefully considered, and characterized by a smaller degree of complexity in terms of structure than written language (Miller and Weinart 2009).

## Present Study

The present study was designed to explore the potential role of linguistic abstraction in people’s perceptions of gender in fairly naturalistic language. Although some words through which linguistic abstraction manifests itself are stereotypically, and sometimes even explicitly gender-linked, the abstraction dimension also underlying the use of interpersonal verbs and adjectives would represent a more subtle way of indicating gender. Simultaneously, the study was intended to provide some insight into the more general effects of linguistic abstraction in natural language, particularly as a potential means of conveying stereotypic content in interpersonal discourse. Whereas linguistic abstraction was previously found to explain (i.e., mediate) much of the effect of expectation conformity on recipients’ dispositional attributions about groups or individuals, it may also alter, or moderate, the effect of information pertaining to other attributes not used as a reference point for linguistic abstraction on recipients’ inferences. As discussed, linguistic abstraction may also transmit meaning directly, considering the two distinct types of inference (i.e., dispositionality and event instigation) it can elicit.

In contrast to previous research into the effects of linguistic abstraction, more natural, spoken language elicited in Experiment 1 provided the basis for text stimuli to be interpreted in Experiment 2. We asked speakers to tell stories about their romantic partner and best friend, expecting that in most cases one of them would be a woman and the other a man (see the Appendix for an example of a story told about a female and male protagonist). Stories were requested in a manner that identified individuals by their relationship to minimize the salience of gender and hopefully avoid cueing gender stereotypes. Instead of asking speakers to tell gender-stereotypical or atypical stories, the stories were coded retrospectively based on their consistency with gender-role norms. Participants in Experiment 2 read transcripts of these stories and judged the gender of the protagonist with explicit linguistic gender cues removed. These gender judgments acted as a more specific measure of dispositionality, or person-versus-situation attributions. Through regression analysis, we were then able to examine the role of linguistic abstraction in recipients’ perception of the protagonists’ gender.

## Experiment 1

Experiment 1 was designed to elicit spoken language by participants about female and male individuals to be used as stimuli in Experiment 2. Participants were asked to tell stories about their romantic partner and best friend. A secondary aim of the first experiment was to explore these stories for linguistic gender indices, focusing on the linguistic abstraction of interpersonal verbs and adjectives, which could influence recipients’ perceptions of the protagonists in Experiment 2. Consistent with the possibilities discussed in the introduction, we examined if the LEB or either the dimension of dispositionality or event instigation may influence participants’ use of linguistic abstraction systematically between descriptions of female and male close others. In particular, we wanted to explore whether or not linguistic abstraction would vary depending on the gender of the close other and/or the level of gender stereotypicality of the event described. For this purpose, close-other depictions were classified into stereotypical, neutral, and atypical, relative to masculine or feminine gender-role norms and depending on the close other’s gender.

If the LEB leads participants to describe close others of one gender in more abstract language than the other, this effect should be mediated by the context’s level of gender stereotypicality (i.e., masculine, feminine, or neutral). Precisely, the effect of close-other gender on linguistic abstraction should be non-significant, or at least weaker, after controlling for the stories’ level of gender stereotypicality. The LEB holds that linguistic abstraction varies depending on how stereotypical the information communicated is for the group or person being described. As discussed, stereotypical information is described more abstractly than atypical information. Differences in the extent to which men and women are described in gender-stereotypical ways could, therefore, elicit systematic differences in the level of abstraction used in descriptions between these two groups. A direct effect of close-other gender on linguistic abstraction, unmediated by the level of gender stereotypicality, would suggest that the LEB does not explain this effect. Rather, it would be indicative of a gender stereotype implicit in either of the two inferences linked to linguistic abstraction: dispositionality or event instigation.

Gender-linked adjectives may also influence the inferences made by the recipients in Experiment 2 about the gender of the person described. Since they fall into one of the categories of linguistic abstraction (the most abstract one), they represent a potential confound in regards to the aims of the experiment, given the focus on gender. Therefore, we also examined the natural occurrence of gender-linked adjectives in the close-other descriptions in this experiment, categorizing them based on their association with traditional gender stereotypes and gender-linked personality traits.

## Method

### Participants

Twenty-one undergraduate and seven graduate students, ranging in age between 18 to 41 years, were recruited from various disciplines at two eastern Canadian universities. The majority of participants (82 %) were between 21 and 29 years old. Eighteen of the participants were female and the other ten were male. Both graduate (2 male, 5 female) and undergraduate students (8 male, 13 female) were recruited to provide a greater range of exposure to egalitarian values, which can be expected to vary with the level of education. Even though such education-based exposure may influence the amount of overt sexist language, it is less clear that it will influence more subtle gender distinctions in language. As an incentive, participants’ names were entered into a drawing for a gift certificate.

All participants were in a close relationship for at least one year with both their best friend and romantic partner, who had to be of different sexes. Data from two additional participants (one female, one male) were not included, as their romantic partners and best friends were of the same sex and, therefore, their use of language to describe female and male close others could not be compared. One male participant’s romantic partner was of the same sex but his best friend was of the other sex. His data were included in the results reported as they met the inclusion criterion of having a best friend and a romantic partner of different sexes. Also, including this case did not affect the observed patterns and significance of *p* levels. The length of relationships ranged from 1 to 23 years, with a mean length of 4.79 years (*SD* = 3.17) for romantic partners and 11.26 years (*SD* = 5.59) for best friends.

### Procedure

Participants were asked to tell stories about their best friend and romantic partner (as opposed to a female and male close other), thereby eliciting one story of the other sex and one of the same sex. A female experimenter interviewed each participant individually.^2^ Before the interview, participants were informed that they would be asked to tell a story about their best friend and romantic partner that is a good example of the type of person they are. Hence, they had time to think about appropriate stories to tell. The order in which participants talked about their romantic partner and their best friend was counterbalanced by gender. Half of the participants of each gender talked about their romantic partner first, whereas the other half talked about their best friend first. The experimenter guided each participant through the interview by asking the following questions, first about the romantic partner or the best friend and then the same questions about the other person:

- Question 1: “How long have you known your partner/best friend?”
- Question 2: “Tell me what your best friend/partner is like. List a few of their qualities.”
- Question 3: “How did you meet your partner/best friend?”
- Question 4: “Tell me a story about your romantic partner/best friend that is a good example of the type person they are.” If necessary: “Can you think of a particular instance as an example of (giving one of the qualities listed in Question 2)?”

The purpose of Question 2 was to warm up each participant, to elicit some adjectives for later comparison, and to provide qualities to prompt the participant if necessary in Question 4. Question 3 was asked to elicit a more gender-neutral story, whereas Question 4 was more open-ended. The session took approximately 20 min was videotaped for later transcription.

### Scoring.

Two independent coders (one female, one male) scored all transcriptions of the interviews. Interpersonal verbs and adjectives pertaining to the close other of the story only (i.e., the romantic partner or best friend) were identified. To look at the more overt referencing of gender, total numbers of the different adjective types (i.e., feminine, masculine, and neutral) were derived from the answers to both the stories and the request to list qualities of the close others (i.e., Questions 2, 3, and 4). For the more subtle references to gender, a linguistic abstraction score was obtained from the stories told about the best friend and romantic partner only (i.e., Question 3 and 4). Frequently, more than one story was told for Question 4 and even sometimes for Question 3, in which case all stories were examined and included within the different linguistic aggregates. To be able to examine linguistic abstraction in relation to the level of gender stereotypicality, separate abstraction scores were also derived for each story. Only verbs and adjectives associated with a close other were included in the scoring where the close other was a sentence subject. Participants narratives consistently depicted close others as the sentence subject.

### Gender-linked adjectives

Adjectives were classified as either masculine (e.g., aggressive, dominant), feminine (e.g., *affectionate, emotional*), or neutral (e.g., *happy, jealous*). Available classifications of gender-linked adjectives (Bem 1974; Williams and Bennett 1975) facilitated this part of the coding procedure, though the lists were not exhaustive for the adjectives encountered. Nevertheless, inter-rater agreement between the male and female rater was perfect (κ = 1.00). Totals of the three adjective types were calculated.

### Linguistic abstraction

The *linguistic category model* (Semin and Fiedler 1988, 1991) classifies interpersonal verbs and adjectives into categories, with increasing levels of abstraction: descriptive action verbs (e.g., *hit, yell, walk*), interpretive action verbs (e.g., *help, tease, avoid*), state action verbs (e.g., *surprise, amaze, anger*), state verbs (e.g., *admire, hate, appreciate*), and adjectives (e.g., *honest, reliable, aggressive*). Thus, descriptive action verbs represent the most concrete word category, and adjectives represent the most abstract word category. Consistent with the “Linguistic Category Model Manual” (Coenen et al. 2006), each category was weighted by assigning a numerical value to it. Descriptive action verbs were assigned a score of 1, state verbs by a score of 3, and adjectives by a score of 4. Interpretive action verbs and state action verbs represent the same level of abstraction and were thus collapsed in one category, which was denoted by a score of 2. The inter-rater reliability was near perfect (κ = 0.98). An average abstraction score is obtained by adding all scores and dividing the sum by the number of coded items, giving a possible range of 1 (very concrete descriptions) to 4 (very abstract descriptions).

### Level of gender stereotypicality

All stories were rated in terms of how stereotypical (n = 47), neutral (*n* = 60), and atypical (*n* = 10) they are, relative to the gender-role norms pertaining to the close other’s gender. Again, the available classifications of gender-linked adjectives (Bem 1974; Williams and Bennett 1975) were used to help facilitate judgment and provide a common reference point for the two raters. The raters were asked to judge the story as a whole in regards to the gender stereotypicality of the close other’s behavior. An equal number of male and female close others were described in gender-neutral and atypical contexts. Of stories classified as stereotypical, 27 described a male close other. Again, inter-rater agreement was high (κ = 0.95).

## Results

The length of the acquaintanceship with the romantic partner and best friend was not related to any of the dependent measures: totals of masculine, neutral, and feminine adjectives, as well as linguistic abstraction scores (*p* > 0.05). Therefore, this variable was not included in further analyses. As the number of graduate students recruited was small, data were also collapsed across level of education. Although both women and men share stereotypical beliefs about gender-role behavior (Williams 1982), participant gender was included in the analyses to control for any gender-of-speaker effects.

### Gender-linked adjectives

Table 1 displays the means and standard deviations for the different linguistic measures across narratives of female and male close others by female and male speakers. A 2 (speaker gender) x 2 (close-other gender) x 3 (adjective type) mixed-design ANOVA compared participants’ use of masculine, feminine, and neutral adjectives to describe their female and male close other. Mauchly’s test indicated violation of sphericity, *χ* ^2^(2) = 26.05, *p* < 0.001. Hence, the degrees of freedom were corrected using Greenhouse-Geisser estimates of sphericity (ε = 0.61). A significant main effect appeared for adjective type, *F*(1.21, 52) = 99.34, *p* < 0.001, partial η^2^ = 0.79. Post hoc analyses indicated that participants produced significantly more neutral adjectives in comparison to feminine and masculine adjectives when discussing both men and women (*p* < 0.05). There were no other significant effects.

**Table 1:** Experiment 1: Means and Standard Deviations for Totals of Adjective Types and Linguistic Abstraction Scores as a Function of Close-Other Gender and Speaker Gender (N = 28)

Close-other gender	Male speakers		Female speakers		Total	
	*M*	*SD*	*M*	*SD*	*M*	*SD*
Adjective types						
Masculine adjectives						
Male	1.60	2.50	1.28	1.23	1.39	1.75
Female	1.30	1.57	0.50	0.79	0.79	0.17
Feminine adjectives						
Male	0.50	0.71	1.22	1.63	0.96	1.40
Female	1.20	0.92	0.72	1.18	0.89	1.10
Neutral adjectives						
Male	7.90	5.26	9.00	5.12	8.61	5.10
Female	6.70	4.08	6.56	3.11	6.61	3.41
Linguistic abstraction						
Male	2.69	0.36	2.51	0.38	2.57	0.37
Female	2.38	0.41	2.39	0.49	2.38	0.45

Linguistic abstraction scores have a possible range of 1 (very concrete descriptions) to 4 (very abstract descriptions)

### Linguistic abstraction

To examine participants’ use of interpersonal verbs and adjectives, a 2 (speaker gender) x 2 (close-other gender) mixed-design ANOVA was conducted on participants’ linguistic abstraction scores. There was a significant main effect for close-other gender, *F*(1, 26) = 5.35, *p* = 0.03, partial η^2^ = 0.17, indicating that participants’ abstraction scores were significantly higher for their descriptions of male close others (*M* = 2.57, *SD* = 0.37) compared to their descriptions of female close others (*M* = 2.38, *SD* = 0.45).

Next, it was examined whether the LEB might explain the observed relationship between close-other gender and linguistic abstraction. In particular, we aimed to test whether gender stereotypicality mediates this relationship, with male close others being described more abstractly than female close others due to men being depicted in stereotypical contexts more often. As the stereotypicality of the story was a nominal variable, a chi-square test was used to examine the relationship between the independent variable (gender) and the potential mediator (gender-stereotypicality). No significant relationship was found, *χ* ^2^(2, *N* = 117) = 0.63, *p* = 0.73. Hence, participants did not describe one gender in more stereotypical contexts than the other.

A last 2 (speaker gender) x 2 (close-other gender) x 3 (gender stereotypicality level) between-design ANOVA was conducted on the stories’ linguistic abstraction scores, to explore if there was a relationship between the possible mediator and the dependent variable (i.e., whether stereotypical stories were described using more abstract language). A linguistic abstraction score was not derived for 27 stories, as they contained a limited number interpersonal verbs and adjectives associated with the protagonist or none at all. This analysis was therefore based on the data of 90 stories. The main effect for close-other gender was replicated (i.e., stories describing male close others contained more abstract language), *F*(1, 78) = 6.68, *p* = 0.01, η^2^ = 0.08, but no other significant main effects or interactions emerged. In total, the level of gender stereotypicality did not mediate the relationship between close-other gender and linguistic abstraction, as it was unrelated to either of these two variables. The final step of the mediation analysis (i.e., examining if the association between gender and linguistic abstraction remains with gender stereotypicality held constant) was therefore redundant.

## Discussion

The transcripts generated in the present experiment were examined for implicit gender indices manifested in the use of interpersonal verbs and adjectives. Participants did not make overt gender distinctions in terms of their use of gender-linked adjectives, which were unrelated to the gender of their close others. This finding decreases the likelihood of gender-linked adjectives confounding the effects in Experiment 2. As gender-linked adjectives represent a more overt way of indicating gender, participants may have been more careful about their use to avoid appearing sexist, particularly as university students. This possibility may also contribute to the significantly higher number of neutral, rather than gender-linked adjectives in their descriptions of female and male close others. Moreover, there was a possible floor effect for masculine and feminine adjectives, providing additional evidence that people might be more careful about making overt gender distinctions.

Other factors to consider may indicate that participants were not trying to control their language. First, there are many more neutral than gender-linked adjectives with which to describe individuals. Second, people are more likely to see familiar people as individuals, rather than in stereotypical ways, as is the case with strangers (Kunda and Thagard 1996). Lastly, a distinction can be made between explicit and implicit stereotyping. Whereas explicit gender stereotype beliefs are more conscious and may be expressed in overt terms, such as gender-linked adjectives, implicit stereotype beliefs need to be assessed less directly (Greenwald and Banaji 1995; Greenwald et al. 2009).

The results did provide evidence for more subtle gender distinctions via linguistic abstraction, as participants used significantly more abstract language to describe male close others and more concrete language to describe female close others. The LEB does not seem to account for this effect; stories of male close others were no more gender-stereotypical than stories of female close others, and story gender stereotypicality (relative to the gender of the story subject) had no effect on linguistic abstraction. As the level of gender stereotypicality was unrelated to linguistic abstraction, there was also no evidence that the LEB reflected the consistency of close-other descriptions with gender-role norms (i.e., individual differences).

It must be remembered that the LEB has typically been studied at the sentence level, whereas we focused on stories. Thus, linguistic abstraction scores were averages of the occurrence of weighted interpersonal verbs and adjectives in a story, as used by Wigboldus et al. (2000, 2006). In contrast to Wigboldus et al. (2000), who requested stories based on their conformity to group norms, we did not ask participants to describe their close others in expected or unexpected events. These differences may help explain the non-significant effect of the level of gender stereotypicality on linguistic abstraction, which is likely influenced by several factors beyond gender-role conformity in natural language (e.g., conformity to personal, social, and other group norms). In the present study, linguistic abstraction may be foremost related to how close others’ behavior in the described events conformed to their typical behavior, or personality, rather than gender-role norms. The reason is that people are less likely to view familiar people in stereotypical ways (Kunda and Thagard 1996).

The gender effect on linguistic abstraction is most likely accounted for by a gender stereotype implicit in one of the two distinct types of inference associated with linguistic abstraction. The present experiment was not designed to assess their relative impact on linguistic abstraction. Nevertheless, since male close others were described in more abstract language than female close others, a stereotype associated with the event instigation factor is an unlikely explanation for this effect. According to this inference, abstract language depicts the protagonist as passive, in contrast to concrete language, which assigns the protagonist a more active role. However, traditional gender stereotypes portray women as being more passive than men and more likely to react to situations, rather than to be the causal agent; men are more likely to display instrumental traits (e.g., assertiveness, leadership ability; Parsons and Bales, 1955; Ruble 1983). The alternative explanation is a gender stereotype linked to the dispositionality factor, specifically that men’s behavior is more predictable/stable than women’s behavior because they are more likely to conform to gender roles. This more likely scenario would reflect the direct and positive association between linguistic abstraction and inferred stability (Semin and Fiedler 1988). Overall, people may ordinarily describe men in more abstract language than women, independent of their relative, actual conformity to gender-role norms.

If a stereotype exists that men are more stable in their behavior than women, then a direct association between linguistic abstraction and recipients’ likelihood to judge the story character as male (controlling for overt indicators of gender) would provide converging evidence for the presence of such a gender stereotype. Furthermore, linguistic abstraction may not only account for but also influence the effects of protagonist behavior on recipients’ inferences about the person being described. When communicators were asked to provide person descriptions varying in expectancy (Wigboldus et al. 2000, 2006), linguistic abstraction mediated the effect of the level of expectancy by the speaker on recipients’ ratings of dispositionality; abstract language led recipients to make significantly more dispositional attributions than relatively concrete language. In natural, spoken language, speakers may use linguistic abstraction to indicate a person’s level of conformity to a particular attribute (e.g., age) without the recipient being aware which attribute is used as the reference point. Instead, the recipient may focus on a different attribute mentioned within the description (e.g., personality) and, consequently, make false inferences from linguistic abstraction about this other attribute (e.g., thinking that silly behavior is atypical for the protagonist’s personality rather than for his or her age).

This kind of scenario may be observed when recipients are asked to make inferences about gender in person descriptions, without being aware of the attribute the speaker is using as a reference point for linguistic abstraction. Specifically, linguistic abstraction as it reflects the protagonist’s conformity to some attribute other than gender may moderate the effects of the descriptions’ stereotypicality (i.e., masculine vs. feminine) on recipients’ perception of that person’s gender. It may be used as an indicator for how typical the information is for the protagonist’s gender, whether intended or not. Theoretically, people may be less inclined to perceive a protagonist of an unknown gender who is described in a feminine or masculine context as female or male, respectively, when the language used is relatively concrete. These possibilities were examined in Experiment 2.

## Experiment 2

The second experiment investigated how linguistic abstraction might be related to recipients’ perception of the close others’ gender (hereafter only referred to as *protagonists*). For this purpose, all explicit gender cues of the protagonist and the speaker were removed from the stories. Participants were given the edited transcripts to read and asked to judge how likely the protagonist’s gender is male versus female, using a single continuous scale. The relationship of these gender judgments with linguistic abstraction, the gender stereotypicality of the context, and gender-linked adjectives used was determined. Additionally we wanted to test whether linguistic abstraction had an incremental predictive effect over gender-stereotypical content and gender-linked adjectives. This observation would indicate that linguistic abstraction is directly associated with gender judgments, reflecting a possible gender stereotype. Consistent with the effect observed in Experiment 1, we made the following hypothesis:

- Hypothesis 1: On the gender-judgment scale, participants will be more likely to rate the gender of a protagonist described in relatively abstract interpersonal language as male rather than female. Conversely, linguistic abstraction in protagonist descriptions should correlate negatively with participants’ likelihood of rating the protagonist’s gender as female.

We also examined if linguistic abstraction influences the effect that gender stereotypicality of context had on participants’ gender judgments. Although a moderation effect of this kind can be inferred from the impact of linguistic abstraction on recipients’ inferences (Wigboldus et al. 2000, 2006), instances where linguistic abstraction and conformity to a particular attribute are unrelated and independent, which may well be the case in natural language, have not been investigated. Also, prior investigations required speakers to write and specifically requested stereotypical and atypical descriptions of the person chosen by the participant. Based on the LEB literature, we also hypothesized the following:

- Hypothesis 2: There will be a significant interaction of stories’ linguistic abstraction and gender stereotypicality (i.e., masculine, feminine, or neutral) on participants’ gender judgments: Participants’ gender judgments will be less likely to match the stereotypicality of the story context for stories described at a lower level of abstraction. By contrast, for stories described in more abstract language, participants’ gender judgments will be more likely to correspond to the story context’s gender stereotypicality.

## Method

### Participants

Sixty participants were recruited from introductory psychology classes of an eastern Canadian university. As is typical of the population of students who enroll in introductory psychology, 49 participants (82 %) identified themselves as female and 52 participants ranged in age from 18 to 20 years (87 %). Participants were also predominately Caucasian and middle-class. They participated in exchange for partial course credit.

### Materials and procedure

All of the stories told by speakers during Experiment 1 (*N* = 117 stories) were randomly divided into one of three questionnaires, as there were too many stories for a participant to read through them all. The numbers of stories per questionnaire were 33, 40, and 44, as speakers rather than individual stories were randomized across questionnaires. A check on the gender stereotypicality of story context across the three questionnaires revealed a relatively even distribution, *χ* ^2^(4, *N* = 117) = 1.67, *p* = 0.80. The respective numbers of readers, or judges, per questionnaire were 21, 21, and 18. All explicit gender-identifying language indices for the protagonist and speaker were changed to make the information gender-neutral. For example, the most common changes were to replace proper names with an *X* and pronouns with either an *X* or *he/she*. After reading each story, participants were asked to rate the gender of the protagonist on a 5-point Likert scale, with 1 being *obviously male*, 3 being *could be male or female*, and 5 being *obviously female*. Participants took approximately 45 min to complete a questionnaire.

## Results

The typicality classifications from Experiment 1 were recoded into three types of gender stereotypicality: 1 (*stereotypically male*), 2 (*neutral*), and 3 (*stereotypically female*). The linguistic abstraction scores derived for each story in Experiment 1 (possible range: 1–4) were used in the present experiment. As explained in Experiment 1, a linguistic abstraction score was not derived for 27 stories and, therefore, the main analysis involving linguistic abstraction was based on the data of 90 stories. Linguistic abstractions scores had an average that was near the scale midpoint of 2.5 (*M* = 2.39, *SD* = 0.59) and ranged from 1.25 to 4.00. The role of participant gender and other participant characteristics could not be examined, since the gender-judgment scores of the stories were averages across the participants who read the stories.

### Correlations and preliminary analyses

There was no significant zero-order correlation between linguistic abstraction and gender judgments, *r*(90) = −0.12, *p* = 0.26. In terms of adjectives types, participants’ gender judgments were significantly associated with the total of masculine adjectives, *r*(117) = −0.33, *p* < 0.001, whereas they did not correlate with the total of feminine, *r*(117) = −0.06, *p* = 0.53, or neutral adjectives, *r*(117) = 0.06, *p* = 0.49. Chi-square tests were executed to explore for possible relationships between gender stereotypicality with the other categorical variables of protagonist and speaker gender. This allowed us to evaluate the validity of our gender stereotypicality classifications and assess speaker gender for any confounding effects. These analyses indicated a significant association of gender stereotypicality with protagonist gender, *χ* ^2^(2, *N* = 117) = 23.79, *p* < 0.001, but not with speaker gender, *χ* ^2^(2, *N* = 117) = 1.85, *p* = 0.40. Male protagonists were more likely to be depicted in masculine contexts (*n* = 27) than female protagonists (*n* = 5), whereas female protagonists (*n* = 20) were more depicted in stereotypically feminine stories than male protagonists (*n* = 5). The same numbers of female (*n* = 30) and male (*n* = 30) protagonists were depicted in gender-neutral contexts.

Table 2 shows the descriptive statistics for participants’ gender judgments as a function of protagonist gender and gender stereotypicality. A 2 (protagonist gender) x 2 (gender stereotypicality) between-design ANOVA on gender judgments revealed a significant main effect for gender stereotypicality, *F*(2, 111) = 28.30, *p* < 0.0001, partial η^2^ = 0.34. Bonferroni-adjusted pairwise comparisons revealed significant differences between all types of gender stereotypicality (*p* < 0.001). Specifically, stereotypically feminine stories had significantly higher scores (*M* = 3.58, *SD* = 0.57) indicating that the protagonist was rated as more likely to be a woman compared neutral (*M* = 2.80, *SD* = 0.52) or male stereotypical stories (*M* = 2.13, *SD* = 0.49). Likewise, male stereotypical stories had significantly lower judgment scores, indicating that, on average, participants rated the protagonists as more likely to be men. Repeating the analysis with speaker gender in place of protagonist gender revealed no additional main or interaction effects. One-sample *t* tests showed that the gender-judgment scale means were significantly different from the scale midpoint of 3 for all story types: stereotypically male, *t*(31) = −10.00, *p* < 0.0001, neutral, *t*(59) = −3.05, *p* = 0.003, and female stories, *t*(24) = 5.09, *p* < 0.0001.

**Table 2:** Experiment 2: Means and Standard Deviations for Gender Judgment Scores as a Function of Stories’ Protagonist Gender and Gender Stereotypicality (N = 117)

Gender stereotypicality^a^	Male protagonists		Female protagonists	
	*M*	*SD*	*M*	*SD*
Stereotypically male	2.07	0.40	2.45	0.81
Neutral	2.65	0.48	2.94	0.52
Stereotypically female	3.62	0.61	3.57	0.58

Gender judgment scores range from 1 (*obviously male*) to 5 (*obviously female*)

^a^Referring to the story context.

### Regression and moderation analysis

Table 3 shows the results of a simultaneous regression analysis predicting gender judgments. The purpose of this analysis was to examine whether linguistic abstraction (a) predicts incremental variance in participants’ gender judgments over protagonist gender, context gender stereotypicality, and masculine adjectives (as neutral and feminine adjectives were unrelated to gender judgments, they were excluded here to avoid compromising the power of the analysis), and (b) moderates the effects of gender stereotypicality on gender judgments. While the overall model accounted for 60 % of the gender-judgment variance, *F*(5, 84) = 25.38, *p* < 0.0001, *R* ^2^ = 0.60, the only significant predictors were protagonist gender and the interaction term of gender stereotypicality and linguistic abstraction. The significant change in *R* ^2^ explained by the interaction was 0.03 (*F* = 7.20).

**Table 3:** Experiment 2: Regression Analysis Summary for Story Variables Predicting Participants’ Gender Judgments (N = 90)

Variable	B	*SE B*	β	*t*	*p*
Protagonist gender	0.23	0.07	0.30	3.53	0.001
Total masc. adjectives	−0.10	0.06	−0.13	−1.62	0.109
Gender stereotypicality	−0.31	0.35	−0.30	−0.89	0.377
Linguistic abstraction	−0.47	0.29	−0.36	−1.59	0.115
Gender Stereotypicality x Linguistic Abstraction	0.39	0.15	0.92	2.68	0.009

Gender judgment scores range from 1 (*obviously male*) to 5 (*obviously female*). For protagonist gender, male protagonists were coded 1 and female protagonists were coded 2. For gender stereotypicality, male-stereotypical stories were coded 1, neutral stories were coded 2, and female-stereotypical stories were coded 3. Linguistic abstraction scores have a possible range of 1 (very concrete descriptions) to 4 (very abstract descriptions)

**Fig. 1:**
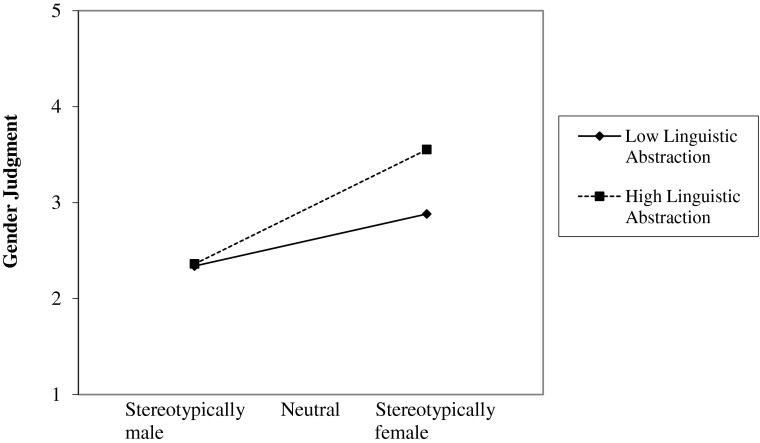
Experiment 2: Gender judgment scores, ranging from 1 (*obviously male*) to 5 (*obviously female*), as a function of stories’ gender stereotypicality and linguistic abstraction, controlling for protagonist gender and the total of masculine adjectives

Figure 1 shows participants’ gender judgments as a function of gender stereotypicality and linguistic abstraction, controlling for protagonist gender and masculine adjectives. The graph indicates that gender stereotypicality had a lesser impact on gender judgments for stories described in more concrete language. To probe the interaction, the Johnson-Neyman regions of significance method was used, specifically to determine the point at which linguistic abstraction no longer inhibits the effect of gender stereotypicality on gender judgments. This analysis showed that gender stereotypicality had no significant effect up to a linguistic abstraction level of 1.55. Thus, it seems that concrete language compromised participants’ certainty that stereotypically masculine and feminine stories really had male and female protagonists, respectively. The figure also indicates that linguistic abstraction did not influence gender judgments for stereotypically masculine stories. Indeed, a simple slopes analysis (Aiken and West 1991) confirmed this observation, β = −0.08, *t*(5, 84) = −0.46, *p* = 0.64.^3^ Linguistic abstraction did, however, have an impact in both neutral, β = 0.31, *t*(5, 84) = 3.04, *p* = 0.003, and feminine stories, β = −0.71, *t*(5, 84) = 3.68, *p* < 0.001, which were significantly more likely to be rated as having a female protagonist when linguistic abstraction was high.

## Discussion

The present experiment examined if linguistic abstraction (a) is directly associated with the perceptions of the protagonists’ gender in spoken language, and (b) moderates the effects of context gender stereotypicality on gender judgments. The results confirm our gender stereotypicality classifications from Experiment 1. Participants rated protagonists of female-stereotypical stories as more likely to be female, whereas protagonists of male-stereotypical stories were rated more likely to be male. In a preliminary analysis of variance, protagonist gender did not have a significant incremental effect on participants’ gender judgments over gender stereotypicality and did not interact with gender stereotypicality. Protagonist gender was only significant in the regression analysis, as the effects of gender stereotypicality were moderated by linguistic abstraction and possibly also affected by the inclusion of masculine adjectives. These initial results suggest that participants based their gender judgments largely on the overall story context, also supporting our decision to code the stories’ context for gender stereotypicality rather than isolated behaviors of the protagonists.

Based on the finding in Experiment 1 that men were described using more abstract language than women, it was hypothesized that participants in Experiment 2 might use linguistic abstraction alone to help judge the gender of the story protagonists. However, linguistic abstraction did not predict incremental variance in gender judgments, let alone correlate significantly with gender judgments in the expected direction. The results, therefore, do not provide converging evidence for a gender stereotype directly implicit in linguistic abstraction, either in the dispositionality or event-instigation factor. A possible explanation is that, unlike the speakers, recipients were specifically cued for gender. Consequently, they may have directed their attention to a multitude of gender indices, many of which may be heuristically more reliable and convenient than linguistic abstraction.

Although linguistic abstraction by itself seemed to have no independent effect on the perception of gender, it interacted with gender stereotypicality to influence gender judgments. This result provides general support for Hypothesis 2, indicating that linguistic abstraction moderated the effects of gender-stereotypical content on participants’ inferences. However, there were important differences between the predicted and observed structure of the interaction. An unpredicted observation is that, for stories described in very concrete language, participants seemed to be less certain that the gender of the protagonists matched the gender stereotypicality of the stories’ context. This result is in line with Hypothesis 2 and research demonstrating that individuals use linguistic abstraction to indicate certainty about what a person is like and make dispositional inferences (Wigboldus et al. 2000, 2006). On the other hand, this observation was not explicated in Hypothesis 2, as we did not expect linguistic abstraction to have such a strong effect on recipients’ perceptions, essentially leading them to question whether the story is a genuine depiction of the protagonist.

Consistent with the predicted interaction, linguistic abstraction was related to participants’ ratings of stereotypically feminine stories. It seemed to influence recipients’ inferences from the content of stereotypically feminine stories about the gender of the protagonist, with concrete language compromising their certainty that the protagonist was female. In fact, as Fig. 1 shows, stereotypically feminine stories described in concrete language were more likely to be judged as having a male protagonist. While this observation is consistent with Hypothesis 2, it was only made for stereotypically feminine stories; linguistic abstraction did not influence participants’ gender judgments for protagonists of stereotypically masculine stories. Overall, concrete language seems to influence people’s perception of information that is specifically female-stereotypical, indicating that linguistic abstraction is used differentially in making gender-linked inferences.

Men are considered the “default” gender when gender is not explicitly specified (Angier 2000). This is evident in myriad ways, such as in the historic convention of the use of the generic *he* in writing (Angier 2000). Therefore, in hindsight it is not particularly surprising that participants would judge protagonists in gender-neutral stories as more likely to be men than women—the average gender-judgment score for neutral stories was significantly below the neutral midpoint, meaning the participants were guessing the protagonist was more likely a man. At odds with this observation is participants’ tendency to judge neutral stories told with relatively concrete language as more likely to have a male, or less likely to have a female protagonist. Perhaps in the absence of gender-stereotypical content, the event-instigation factor of linguistic abstraction influenced participants’ gender judgments. Concrete language ascribes an active role to the protagonist, whereas abstract language can be interpreted as the protagonist being passive, and these traits seem to be respectively associated with traditional stereotypes corresponding to each gender (Parsons and Bales, 1955; Ruble, 1983).

## General Discussion

The purpose of the present study was to explore the possible role that linguistic abstraction may have in people’s perception of gender in spoken language. Experiment 1 was primarily conducted to create the stimuli for Experiment 2, but the stories told by participants were also examined for any gender indices implicit in the use of interpersonal verbs and adjectives. Male protagonists were described in more abstract language than female protagonists, whereas the more overtly gender-linked adjectives were unrelated to protagonist gender. The gender stereotypicality of the stories was also unrelated to protagonist gender and linguistic abstraction, making it unlikely that the gender effect on linguistic abstraction can be attributed to the LEB. It is less conceivable that our chosen methodology explains the non-significant association of the level of gender stereotypicality with linguistic abstraction and protagonist gender; the inter-rater reliability for our gender stereotypicality level ratings was high and the recoded classification did produce the hypothesized effects in Experiment 2. We maintain that the most likely explanation for male protagonists being depicted in more abstract language is a gender stereotype implicit in linguistic abstraction, particularly the dispositionality factor. People may view men’s behavior as more stable and predictable than women’s behavior, but the effect will need to be replicated and the proposed explanation further tested.

The results of Experiment 2 provide much greater certainty concerning the mechanism through which linguistic abstraction was involved in participants’ inferences about story protagonists’ gender. Specifically, linguistic abstraction seemed to influence their certainty about the gender of the protagonist based on the gender stereotypicality of the context, particularly in the cases of stereotypically feminine contexts. Recipients’ gender judgments were more likely to reflect the gender stereotypicality of the story for female-stereotypical stories described in abstract language. This effect is consistent with the dispositionality factor of linguistic abstraction, whereby more abstract interpersonal language is interpreted as meaning greater stability, predictability, and, thus, typicality of information. On the other hand, it remains unclear why for gender-neutral stories relatively concrete, rather than abstract, language reinforced the perception of the protagonist as male. Gender neutral and stereotypically masculine stories were judged more likely to have a male protagonist, with the effect for neutral stories being explainable with the notion that people view male as the default gender.

## Implications

It was reassuring that gender was not evidenced in a more overt fashion through gender-linked adjectives. This pattern may be due to our more educated sample and was possibly influenced by the experimental environment. Yet, the findings suggest that subtle gender distinctions are communicated in an implicit way via linguistic abstraction. Although society has slowly advanced in minimizing its use of explicit and often overtly sexist terms, these findings are consistent with the concept of modern sexism. That is, people continue to make gender distinctions in ways that are implicit, which nonetheless have the potential to influence others’ perception of the information being communicated.

While the results revealed another medium through which modern sexism may be enacted by speakers (i.e., linguistic abstraction), it is uncertain to what extent these gender-based variations in linguistic abstraction shape recipients’ perceptions of the semantic content, as linguistic abstraction had no independent effect on their inferences. Still, recipients used linguistic abstraction as a means to judging the protagonist’s gender for stereotypically feminine, but not masculine stories. Therefore, people may use subtle linguistic features differentially in forming their impression about men and women, which speaks to the persistence of gender-biased processing of information in Western culture. Possibly, these gendered and therefore schematic ways of processing information operate below the level of awareness of the speaker or the recipients.

The moderating effect of linguistic abstraction also has implications for understanding its role in everyday language. Previous research has shown that linguistic abstraction can explain how expectation conformity is indicated, and the inferences drawn by recipients (e.g., Wigboldus et al. 2000, 2006). The present findings suggest that linguistic abstraction may alter the recipient’s interpretation of information pertaining to person attributes other than those used as a reference point for linguistic abstraction. There are many attributes relative to which linguistic abstraction can be used to indicate expectancy or stereotypicality—an abstract differentiation is that of personal and group norms. Nevertheless, linguistic abstraction as it relates to a particular attribute in natural language can influence the effect of other stereotypical information, unrelated to linguistic abstraction, on recipients’ inferences pertinent to that information.

In spite of the wealth of research demonstrating gender differences in spoken language (see the meta-analytic review by Leaper and Ayres 2007), we had no reason to predict gender differences in the use of interpersonal verbs and adjectives. Both women and men share stereotypical beliefs about gender (Williams 1982) and together construct gender. Indeed, female and male participants did not differ in their use of gender-linked adjectives or linguistic abstraction to describe story protagonists. We minimized the perception of other-sex individuals as out-group members and in stereotypical ways by requesting the stories based on their relationship, rather than cueing for gender, and about close others, as opposed to strangers, respectively. Therefore, our results in conjunction with the previous research on the linguistic intergroup bias (e.g., Maass et al. 1995) indicate that the gender of the speaker is only relevant in research on linguistic abstraction when it is salient that the focus is on gender and/or when speakers are asked to describe less familiar people.

## Future Directions

These interesting findings require replication with samples from other populations to ascertain that there is both generalizability and reliability in the observed effects. For example, more research is needed to isolate the reason for the finding in Experiment 1 that men were generally described in more abstract language. A starting point would be to investigate if women violate gender-role norms more often than men (to completely rule out the LEB as a possible explanation) and if people view men’s behaviors as more stable and predictable than that of women. More generally, further research is needed to investigate the use and effects of linguistic abstraction in natural language. It remains relatively uncertain under what circumstances and through which particular mechanisms linguistic abstraction shapes the impressions of the perceiver.

In regards to future research designs, it will be important to address the issue of independence amongst stories when examining their effects on recipients’ inferences. In addition, future studies in this area should also address the implications of recipient gender and the potential role of the gender of the interviewer. People’s language changes depending on the gender of their conversational partner (Fitzpatrick et al. 1995). In fact, language use can even change depending on whether individuals are being interviewed by an experimenter compared to when talking to a friend or in a non-interview setting (see Pasupathi 2001). While the present study examined linguistic abstraction in a much more natural setting compared to previous research, future research on the topic would benefit from taking a more dynamic, contextual approach to determine the extent of its role in communication.

## Appendix

An example of stories told about a male and female close other from Experiment 1, modified by removing explicit gender-identifying information to be read by participants in Experiment 2. X’s at a different university so we don’t see X a whole lot . . but I mean, whenever X is home, X always . . organizes everything. Like X comes home and well, probably the best one, we went on a trip to Montreal . . and before we went, X had . . the whole list of things we’re gonna do. X was like ‘we’re gonna arrive at about this time. So we’ll go, we’ll unpack, . . we’ll go out to dinner here, and then we’ll go downtown then we’ll come home, get up about this time, go and get the continental breakfast at the hotel, and then we’ll go look at this and then after that we’ll go for lunch, and then we’ll go look at this.’ And X had it all planned out, hour by hour, minute by minute. But of course like the plans never go like you want but X’s always so . . optimistic about who’s gonna do what and it’s always gonna go as plan, but it never does. Well I guess the legendary birthday story was that, I think, …. It would have been… four years ago probably because it was the first, I think it was the first birthday we were together. And uh X had a party at his/her house with his/her roommate and uh a couple of people that were invited showed up. And after everybody had a few drinks one of them put a hand in X’s roommate’s face and uh you know my partner is small but is powerful and X flew off the handle and uh this other person was twice as big as X but had them out of the house. And that that’s a legendary story. That’s the jokes we always talk about that story.

## Footnotes

1 It is important to emphasize that the word ‘stable’ or ‘stability’ is used in the literature to refer to the consistency and, thus, predictability of behavior, rather than to denote its quality (as in lability or saneness).

2 We specifically chose to use a female interviewer, as both men and women feel more comfortable disclosing personal information, especially emotion-related information, to a female than male recipient (Snell et al. 1989).

3 To ascertain that the inclusion of masculine adjectives did not explain the non-significant slope for stereotypically masculine stories, we repeated the simple slopes analysis without this variable. However, removing this variable from the analysis did not affect the pattern of the results.
